# Innovative gait robot for the repetitive practice of floor walking and stair climbing up and down in stroke patients

**DOI:** 10.1186/1743-0003-7-30

**Published:** 2010-06-28

**Authors:** Stefan Hesse, Andreas Waldner, Christopher Tomelleri

**Affiliations:** 1 Medical Park Humboldtmühle Berlin, Department Neurological Rehabilitation Charité - University Medicine, 13507 Berlin, Germany; 2 Privatklinik Villa Melitta, Neurological Rehabilitation, 39100 Bozen, Italy; 3 Research Department for Neurorehabilitation South Tyrol, 39100 Bozen, Italy

## Abstract

**Background:**

Stair climbing up and down is an essential part of everyday's mobility. To enable wheelchair-dependent patients the repetitive practice of this task, a novel gait robot, G-EO-Systems (EO, Lat: I walk), based on the end-effector principle, has been designed. The trajectories of the foot plates are freely programmable enabling not only the practice of simulated floor walking but also stair climbing up and down. The article intended to compare lower limb muscle activation patterns of hemiparetic subjects during real floor walking and stairs climbing up, and during the corresponding simulated conditions on the machine, and secondly to demonstrate gait improvement on single case after training on the machine.

**Methods:**

The muscle activation pattern of seven lower limb muscles of six hemiparetic patients during free and simulated walking on the floor and stair climbing was measured via dynamic electromyography. A non-ambulatory, sub-acute stroke patient additionally trained on the G-EO-Systems every workday for five weeks.

**Results:**

The muscle activation patterns were comparable during the real and simulated conditions, both on the floor and during stair climbing up. Minor differences, concerning the real and simulated floor walking conditions, were a delayed (prolonged) onset (duration) of the thigh muscle activation on the machine across all subjects. Concerning stair climbing conditions, the shank muscle activation was more phasic and timely correct in selected patients on the device. The severely affected subject regained walking and stair climbing ability.

**Conclusions:**

The G-EO-Systems is an interesting new option in gait rehabilitation after stroke. The lower limb muscle activation patterns were comparable, a training thus feasible, and the positive case report warrants further clinical studies.

## Background

The annual stroke incidence is approximately 180 per 100.000 inhabitants in the industrialized world [1]. Three months after a stroke, a third of the surviving patients are still wheelchair-dependent, and the gait velocity and endurance are significantly reduced in approximately 80% of the ambulatory patients [2]. Accordingly, the restoration and improvement of walking functions is a primary concern with respect to the aspired social and vocational reintegration.

To achieve this goal, a task specific repetitive training seems most promising [3]. The conventional physiotherapy instead focuses on strengthening and practicing single movements or various neurofacilitation techniques, but these methods do not stress gait practice.

One treatment approach to increase steps number during training sessions is the treadmill training with partial body weight support. [4,5]. However the assignment of human resources for manual assistance in this method is considerable; up to three therapists have to place the paretic limb during the swing phase and to shift the patient's weight onto the stance limb.

Consequently, gait machines followed, either applying an exoskeleton [6-9] (e.g. Lokomat, LOPES, ALEX, AutoAmbulator) or an end-effector principle [10-12] (e.g. Gait Trainer GT I, HapticWalker, LokoHelp). The exoskeleton is equipped with programmable drives or passive elements which flex the knees and hips during the swing phase, whereas with the other principle the feet are placed on foot plates, whose trajectories simulate the stance and swing phases. Clinical trials in stroke patients revealed non-equivocal results for the Lokomat [13] and a consistently superior effect for the GT I [14] with respect to the restoration of gait. A head-to-head comparison of the clinical effectiveness between existing machines is missing. An accelerometry-based biomechanical comparison between the Lokomat and the GT I showed comparable mechanical constrains that may alter leg accelerations and decelerations during stance and swing phases [15].

The currently commercially available gait machines (Lokomat, AutoAmbulator, LokoHelp and GT I) are limited to the repetitive exercise of walking on the floor. Stair climbing up and down, however, is an essential part of everyday's mobility, and recent reports indicated that only 5 to 25% of stroke patients can master one floor at their discharge home from early rehabilitation. [16]. To improve the outcome, the patients should frequently practise stair climbing up and down in line with the task-specific repetitive approach. However the inherent physical effort of the therapists limit the intended intensity, a further burden is the risk of falls on the stairs.

The Haptic Walker [17], an end-effector based robot with fully programmable trajectories, was the first device to additionally enable harness-secured patients the repetitive practice of stair climbing up and down without overstressing therapists. The dimensions and the required high voltage, resulting from the goal to achieve a maximum acceleration of 3,5 g during the push-off and a maximum speed of 5 km/h as during natural gait of healthy subjects [18], limited its clinical utility.

**Figure 1:**
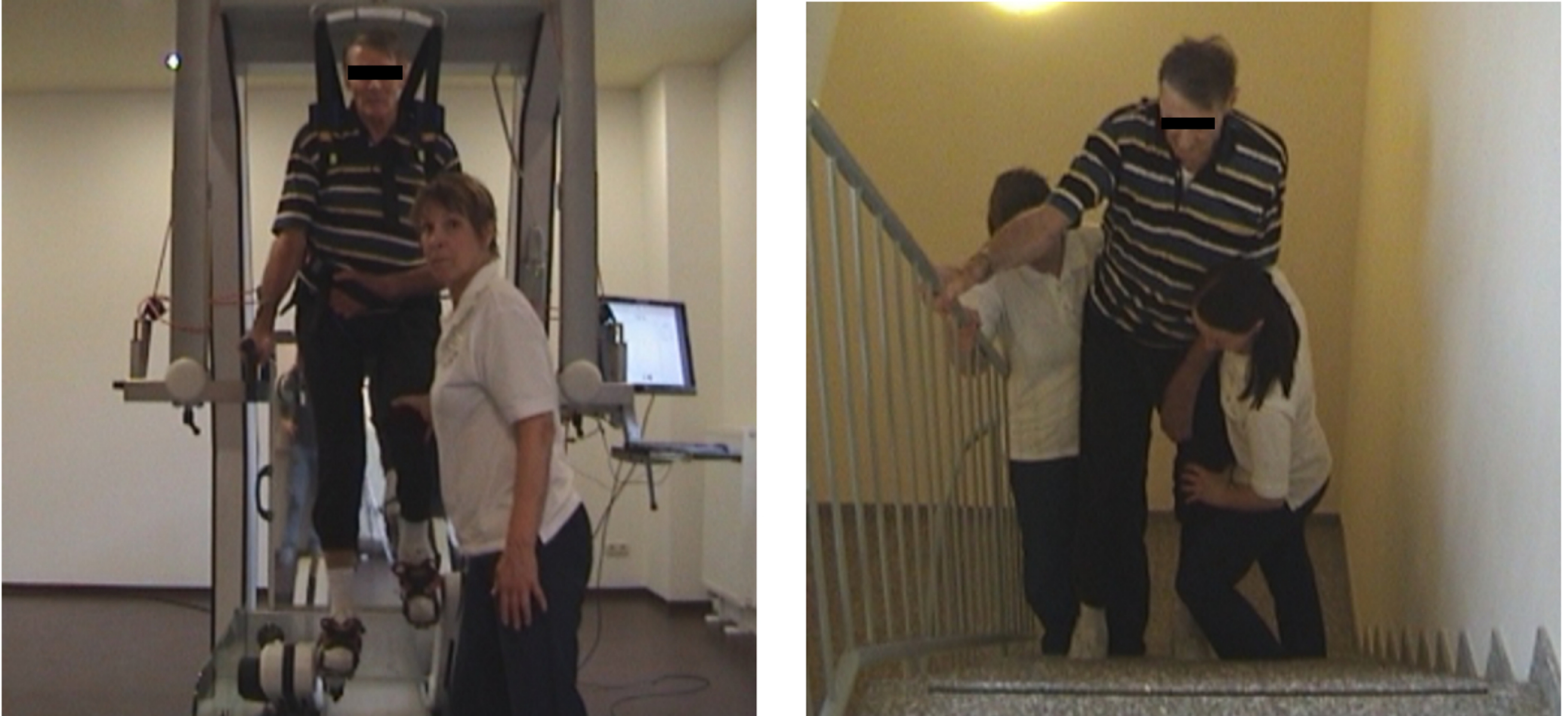
**Wheelchair-bound left hemiparetic patient practising stair climbing**. On the gait robot, the therapist assists the extension of the affected knee (left), the same patient practising stair climbing with the help of two therapists (right).

Accordingly, the present work introduces a newly developed gait robot (G-EO-Systems; EO, latin: I walk) for the treatment of stroke patients (Figure 1). Its specifications included smaller dimensions and an energy supply of 230 V. The main aim of the present study was to compare limb muscle activation patterns of hemiparetic subjects during real and simulated floor walking, and during real and simulated stair climbing up by means of dynamic electromyography. Comparable muscle activation patterns between the real and simulated conditions, and the lack of obviously deviant patterns induced by the gait robot should help to dissipate any fears of the induction of a pathological gait on the machine. The second aim was to demonstrate gait improvement on a single case after a five weeks training with the new machine.

## Methods

### Patients

Six subacute stroke patients participated. They all had suffered a supratentorial ischemia resulting in a right (left) hemiparesis in three cases each, the stroke interval reached from 6 to 14 weeks. Their age was less than 75 years, all of them could walk independently at least a distance of 20 m at a velocity of more than 0.25 m/s, and could climb 10 stairs in an alternate fashion, the use of technical aids or hand rails was allowed. The lower limb spasticity was mild to moderate, the modified Ashworth score (0-5) to assess hip, knee and ankle tonus, did not exceed a value of 2 for any of the joints. All patients understood the purpose and content of the protocol, approved by the local ethical committee, none of them had any other orthopaedic or neurological disease impairing gait, nor an apparent heart failure.

### The device

**Figure 2:**
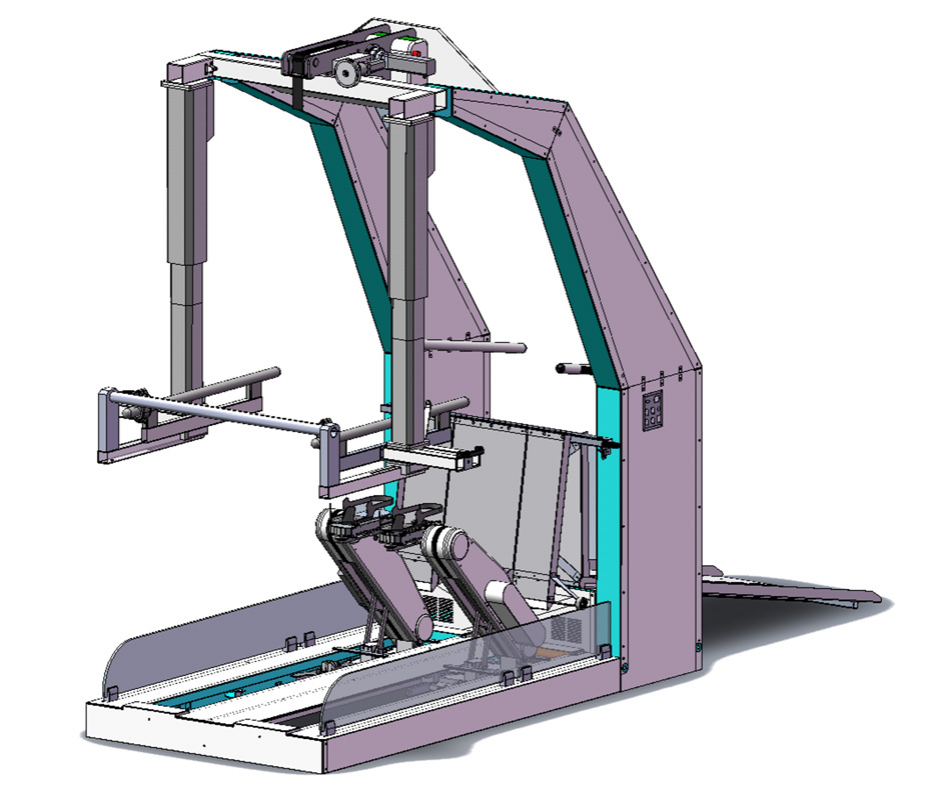
**The G-EO-Systems Robot**. A three-dimensional view of the new gait robot with freely programmable foot plates, the patient lifter, body weight support system, handrails and the ramp.

**Figure 3:**
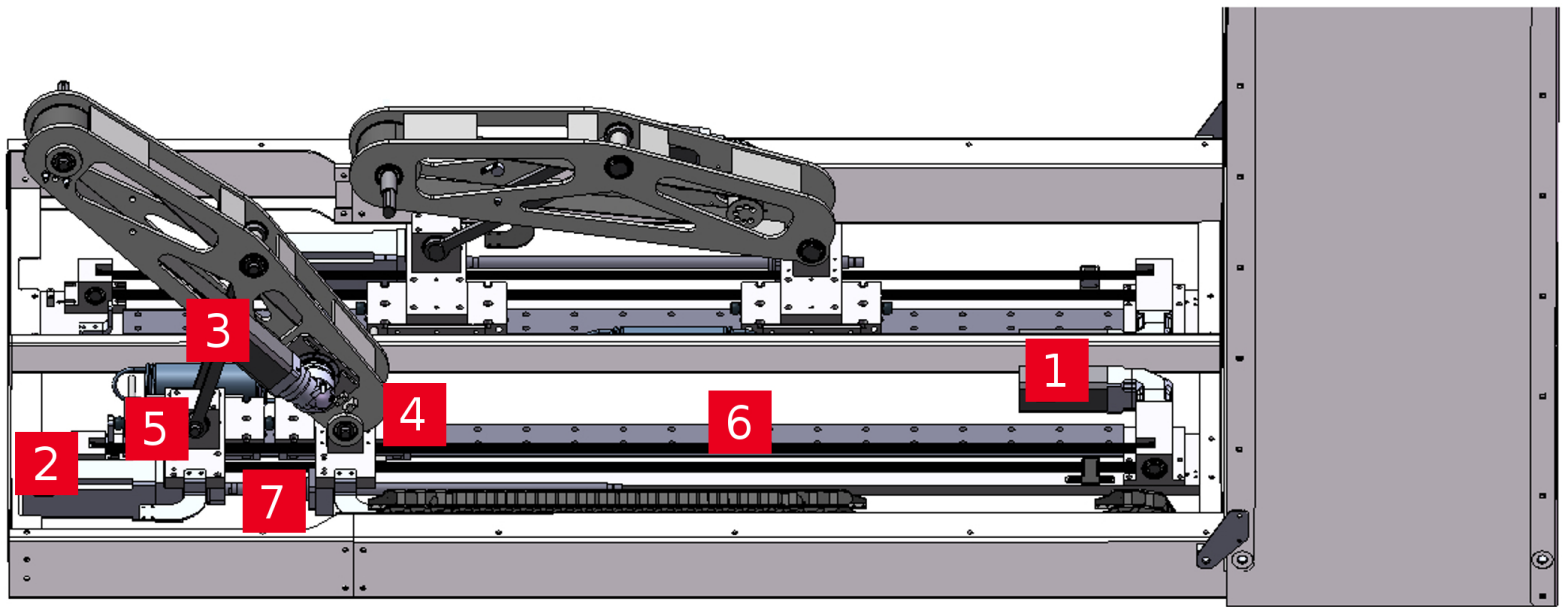
**Three-dimensional sketch of the motion mechanism of the gait robot**. The components are numbered as follows: 1. principal drive, 2. relative drive, 3. drive for the foot angle, 4. principal sledge, 5. relative sledge, 6. linear guide, 7. screw axle.

The device (Figure 2) followed the end effector principle. The harness secured patient stood on two foot plates, whose trajectories were completely programmable. The two foot plates were connected each by a pivoting arm to two moving sledges. The foot's forward motion was given by the movement of the principal sledge, which was connected to the transmission belt of the linear guide (Figure 3). The transmission belt was driven by a 1.500 W servomotor fixed to the back end of the linear guide. The forward and backward excursion of the principal sledge ensured the control of the step length. The mechanic design for the control of the step height was realized implementing the scissor principle. The second sledge on the linear guide moved relatively to the principal sledge. A rod ensured the connection of the relative sledge to the pivoting arm. Nearing the relative sledge to the principal sledge closed the scissor, providing the pivoting arm to get lifted, and vice versa. The servomotor responsible for the relative motion was fixed under the relative sledge and connected to the principal sledge by a screw axle. A third completely programmable 400 W drive was fixed on the arm and transferred the rotation through a transmission belt to an external axle, which was aligned to the ankle, controlling the plantar- and dorsiflexion during the steps.

The motion control of each servomotor was provided by an industrial personal computer, which was coupled to the eight motion controllers responsible for each programmable degree of freedom on the machine, three for each leg and two for the CoM control. All the electronics and the electric parts were placed in the control box in the back end of the device.

The maximal step length corresponded to 55 cm, the maximal achievable angles were ± 90°. The step height in the workspace was 40 cm, allowing the patient to climb a standardized step of 18 cm. The maximal possible gait velocity was 0,6 m/s, corresponding to an acceleration peak of 10 m/s^2^.

The feet were placed in two snowshoe bindings on a steel plate, which was fixed to the basis plate by magnets. The plate loosened in all three directions of the footplate, if a limit momentum of 4 Nm was exceeded. Hand rails at both sides were settable vertically and laterally. The patient's body weight support system was fixed by an aluminium chassis. It consisted of an electrical patient lift system, intended for helping the patients to stand up from the wheelchair, and a drive activating a three roll mechanism. The patent lifter's belt passed through the three rolls mechanism and was attached to patient's harness. The belt got shortened by the mechanism's motion, ensuring the vertical motion of patient's centre of mass (CoM). The two ends of a rope were fixed to patient's harness at hip height to control the lateral motion of patient's CoM. Another drive moved the rope.

A ramp allowed wheelchair access into the device from behind, to be followed by getting in the snowshoes, fastening them, securing the patient lifter's belt to the harness, standing up with the assistance of the therapist, and a last check before starting therapy.

The trajectories of the foot plates during the floor walking condition were taken from healthy subjects' data in the literature, the same applied to the vertical and horizontal movements of the CoM [19,20]. To simulate the stair walking condition up and down, the stair climbing of healthy subjects was assessed with the help of an active marker system based on ultrasound (Zebris). The relevant markers for the determination of the gait trajectories were placed at the following positions: toe, metatarsale V, heel and hip. The trajectory raw data, recorded by the motion capture system, got processed by a filtering block. First processing was a average filtering considering the actual sample and the two subsequent samples. After the average filter, data were filtered by a second order Butterworth low pass filter. The last smoothing procedure was given by a regularization of the jerk [21], the third derivative of the position information.

The data relative to the marker placed on the metatarsale V provided the information for the up- and down movement and the forward-backward movement of the feet. The data of the metatarsale V got transformed into hip relative coordinates by subtracting the coordinates of the hip (marker placed on the trochanter major) to the coordinates of the metatarsale V. By this transformation, the hip was considered as fixed in the forward-backward direction as there is no progression on the floor during the simulated gait on the robot.

The data relative to the marker placed on the heel provided the information necessary to find the inclination of the foot plates. The position coordinates of the metatarsale V were subtracted to the coordinates of the heel. The arcustangens of this arithmetical operation provided the inclination of the foot plates.

The movement of the CoM during stair climbing was that measured with the ultrasound system, the marker was attached to the hip, and confronted with the available data in the literature [22,23].

The graphic user interface (GUI) showed the actual trajectories for any of the conditions on-line, so that the therapist was able to control and to correct it. Changes could be made for step length, step height, the toe off and the initial contact inclination angles of the feet. For a perfect match of the listed trajectory settings, the patient was fixed in the snowshoe bindings in such a way that the marked position of the metatarsale V in the binding corresponded to the patient's metatarsale V. The therapist could further adapt the excursions of the CoM in the vertical and horizontal directions and the relative position of the suspension point with respect to the foot plates. The PC memorized the treatment conditions of each individual patient. The dimensions of the CE-certified machine (medical device directive 93/42/EEC) were 2.800 mm × 1.200 mm × 2.300 mm, the net weight was 850 kg, the power supply was 230 V.

### Intervention

The gait was subsequently analysed during the following four conditions: 1. hemiparetic walking on the floor at self selected speed, 2. simulated walking on the machine at comparable speed, cadence, and stride length in a highly symmetric fashion, 3. stair climbing up for one flight at self-selected pace in alternate fashion, and 4. simulated stair climbing at comparable step rise and cadence.

The 10 m test helped to derive the mean basic cycle parameters to be set on the machine for each individual subject (condition #2). The time needed for a 10 m walking distance at self selected speed was taken with the help of a stop watch, and the number of steps counted. The speed (cadence) was calculated in m/sec (steps/min), the stride length (m) according to the formula: speed divided by twice the cadence. On the machine, the patients not only walked at comparable basic cycle parameters as on the floor, additionally in a highly physiological fashion as the limb-dependent cycle parameters, the gait symmetry, step height, the initial contact and toe off angles were set according to values of healthy subjects of comparable height. The vertical (lateral) movement of the hip was set to 2 cm (2,5 cm) in all subjects.

To set the stair climbing condition parameters (#4), the patients climbed one flight in an alternate fashion, stair by stair. The time needed was taken, to calculate the speed (stairs/min), the normed step rise was 18 cm. The initial contact and toe off angles corresponded to the floor walking trajectory. The vertical (lateral) displacement of the hip was set to 5 cm (2,5 cm). There was no displacement of the trajectory in the forward or backward direction.

### Assessment procedures

Before the actual assessment, the patients practised the conditions several times to get acquainted to, and to derive the parameters for the conditions #2 and #4. If necessary a metronome helped with pacing. Next the machine parameters were set, the patients instrumented, and the four conditions were assessed in a random order within one session. For each condition, the assessment time was 30 seconds, or at least 10 strides.

The gait analysis system (Infotronic) consisted of overshoe slippers of various sizes with 8 force sensors integrated to assess the limb-dependent cycle parameters. Data were collected at 1000 Hz, amplified and memorized by a portable data logger worn by the patient.

The electromyographic activity of seven lower limb muscles of the affected side (Mm. tibialis anterior, gastrocnemius, vastus medialis, vastus lateralis, rectus femoris, biceps femoris, and gluteus medius) were detected by pairs of self-adhesive surface electrodes (diameter 8 mm) following a standardized protocol: the electrodes were attached 2 cm apart on the muscle bellies after a conventional skin preparation (shaving, cleansing, and abrasion of keratinized epidermis). The impedance was checked and kept below 5 kΩ. Signals (sampling rate 1.000 Hz) were pre-amplified with standard preamplifiers of the Infotronic system attached to the limb and memorized by the portable data logger.

### Data analysis

All gathered signals (i.e, foot contacts, electromyographic measurements) were transmitted after the end of each trial to a personal computer and further processed by Infotronic and Matlab software [24]. Cycle parameters were averaged over at least 10 strides.

The electromyographic data were digitally filtered with a first order lowpass filter (cutoff at 500 Hz) and the single steps were determined by the trigger signal provided by the overshoes. The single steps were time normalized to the mean cycle duration set to 100%, and up-sampled using a cubic spline [25,26]. The muscle activation patterns for each stride were then smoothed using a 150-point root-mean-square (RMS) algorithm. After calculating the RMS of the activation patterns, the mean EMG pattern for the gait cycle was generated by averaging all the individual stride cycles taken by the subject during the 30-seconds data collection sequence. No signal processing occurred for the amplitudes of the measured muscles. In a first step, two experienced raters visually checked the muscle activity patterns of each individual subject for any obvious differences across the four conditions. In a second step, the mean onset and offset points of the activation patterns were determined by means of thresholding the EMG envelope. The onset and offset points were subsequently rounded, to lower the accuracy of the measured data down to 5% of the gaitcycle. The mean error of this loss of accuracy was determined to be 1,25% of the whole gait cycle, calculated as the half of the maximum error possible of the rounding process. Statistics (Wilcoxon test, p < 0.05) compared the on- and offset points and duration of activity between conditions #1 and #2, respectively between conditions #3 and #4. Patients with a tonic or absent muscle activity pattern were excluded.

## Results

### Results of the EMG analysis

**Figure 4:**
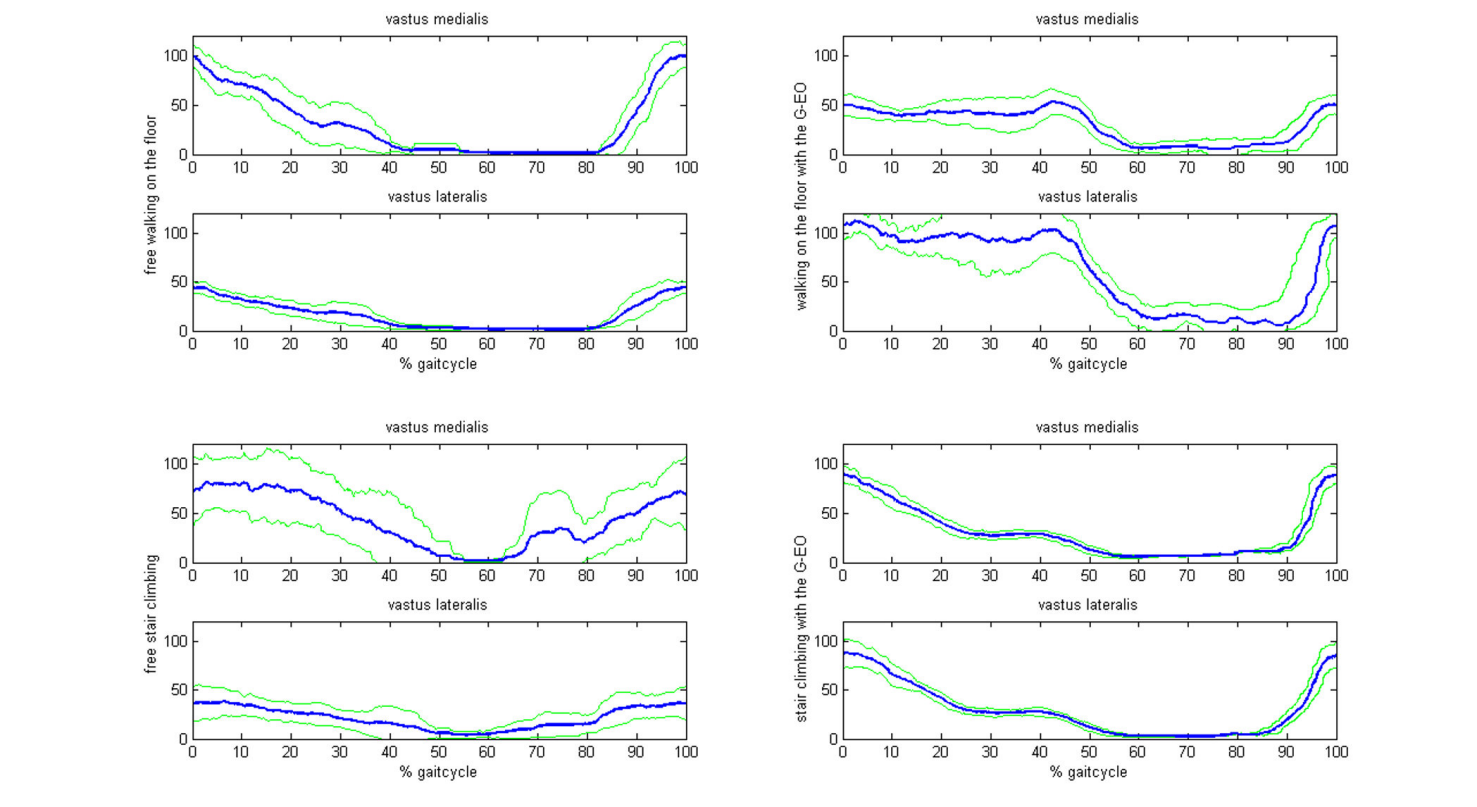
**EMG of the thigh muscles of the affected side in a hemiparetic subject**. The left column shows the activation pattern of the muscle during the real condition, the right column refers to the simulated condition. Note a delayed onset and prolonged activity of the thigh muscles on the machine. The blue lines show the EMG activation pattern of the thigh muscles, the green lines represent the standard deviation of the EMG envelope.

**Figure 5:**
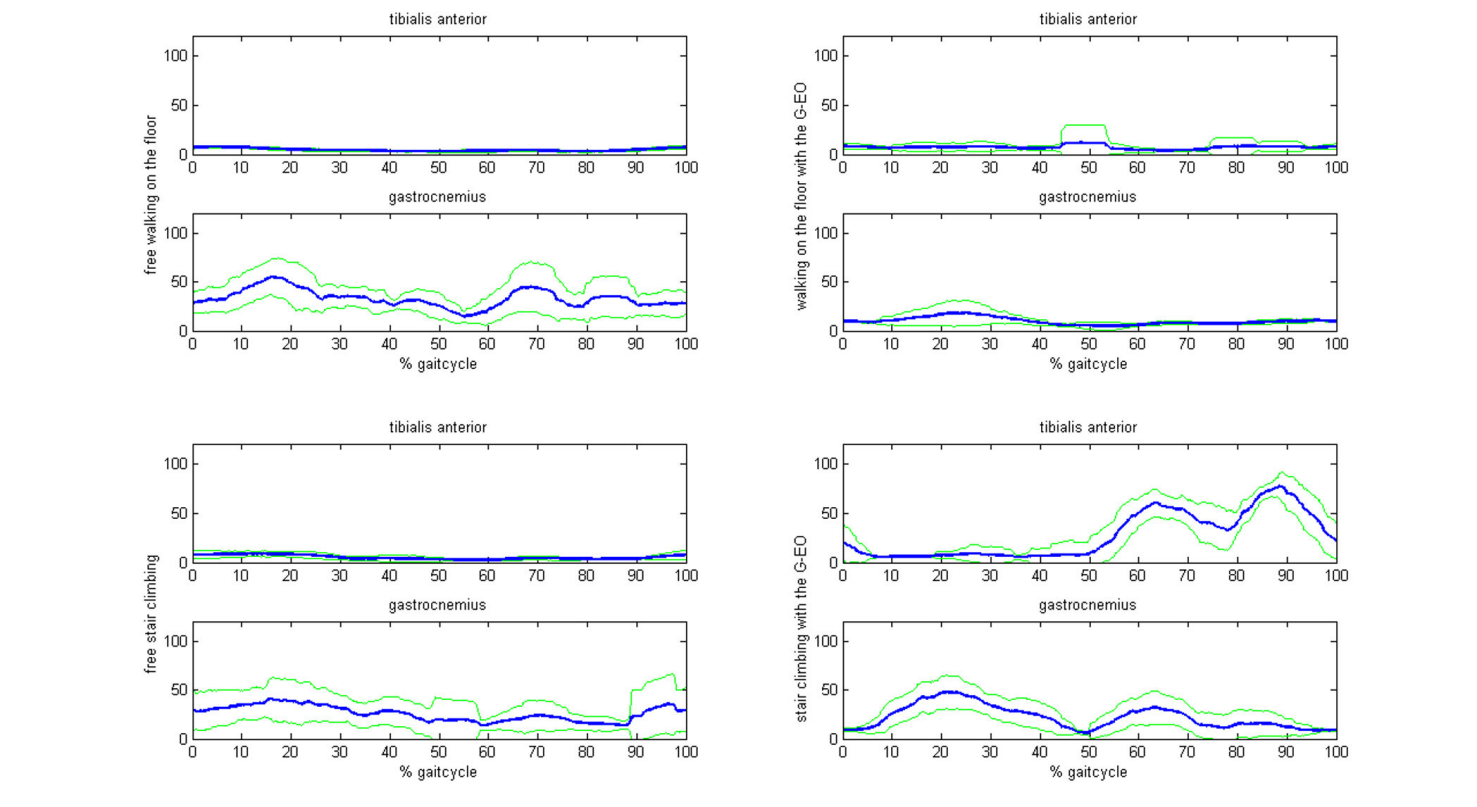
**EMG of the shank muscles of the affected side in a hemiparetic subject**. The left column shows the activation pattern of the muscle during the real condition, the right column refers to the simulated condition. Note the timely correct activation of the Mm. tibialis anterior and the more phasic pattern of the Mm. gastrocnemius on the machine. The blue lines show the EMG activation pattern of the shank muscles, the green lines represent the standard deviation of the EMG envelope.

For the floor walking condition, the pattern of the thigh muscles (Mm. vastus medialis, lateralis, glutues medius) was comparable during the real and simulated conditions across all subjects. Minimal deviations were a delayed onset and a prolongation of the activation of the Mm. vastus medialis, lateralis during the simulated walking (p < 0.05). Instead of the vastus medialis muscle, two subjects more activated the vastus lateralis on the machine (Figure 4). For the shank muscles, deviations became apparent for two subjects. The tibialis anterior muscle remained rather silent during the real and the simulated floor walking, whereas the activity of the gastrocnemius muscle showed a tonic activation pattern during the real and a phasic, but less intense, activation pattern during the simulated walking on the floor (Figure 5). For the remaining four subjects no clear differences became apparent. Table 1 resumes the relevant data of the activation patterns of the shank and thigh muscles for both floor walking conditions.

**Table 1:** Muscle activation data of six hemiplegic patients for the real and the simulated floor walking condition.

			real walking on the floor			simulated walking on the floor		
	tibialis anterior	gastrocnemius	vastus medialis	vastus lateralis	tibialis anterior	gastrocnemius	vastus medialis	vastus lateralis
1	silent	silent	90-10	90-10	silent	silent	90-40	0-25
2	tonic	tonic	80-40	80-40	tonic	tonic	90-50	80-40
3	silent	silent	90-30	90-30	silent	silent	90-35	90-40
4	tonic	tonic	85-40	85-40	40-10	20-55	silent	90-50
5	silent	tonic	85-35	90-30	silent	tonic	80-50	80-50
6	55-20	90-50	80-50	0-70	90-20	15-45	30-60	30-60

The left side of the table shows the data of the shank and thigh muscles relative to the real floor walking condition, the left side of the table shows the data of the shank and thigh muscles relative to the simulated floor walking condition. Note a slight delay in the onset of the mean activation times for the simulated condition in respect to the real condition.

For the stair climbing condition, the activation pattern and the amplitudes of the thigh muscles were comparable during both conditions. For the shank muscles, distinct differences became apparent in three out of the six patients, in the sense that the tibialis anterior muscle was activated in a timely correct fashion (p < 0.05). At the same time, the activation pattern of the gastrocnemius muscle became more phasic. In the other three patients, the activity of both muscles was rather low and tonic and did not differ across conditions. Table 2 shows the relevant data of the activation patterns of the shank and thigh muscles during real and simulated stair climbing.

**Table 2:** Muscle activation data of six hemiplegic patients for the real and the simulated stair climbing condition.

			real stair climbing				simulated stair climbing	
	tibialis anterior	gastrocnemius	vastus medialis	vastus lateralis	tibialis anterior	gastrocnemius	vastus medialis	vastus lateralis
1	silent	silent	tonic	Tonic	silent	silent	tonic	tonic
2	tonic	tonic	90-55	85-55	tonic	tonic	85-50	85-45
3	silent	silent	silent	90-40	silent	silent	90-40	85-45
4	tonic	tonic	90-40	90-50	40-60	5-45	80-50	80-50
5	silent	silent	80-40	80-30	55-95	10-40	85-40	85-40
6	tonic	tonic	90-10	Tonic	50-20	20-55	80-20	90-20

The left side of the table shows the data of the shank and thigh muscles relative to the real stair climbing, the left side of the table shows the data of the shank and thigh muscles relative to the simulated stair climbing. Note a more phasic activation of the shank muscles and the correct activation of the thigh muscles, for what concerns activation time and duration.

### Clinical case report

The patient was a 72 year old male. A supratentorial ischemic stroke in the territory of the right middle cerebral artery had resulted in a severe hemiparesis left with a neglect syndrome. Five weeks after stroke onset, he was able to sit at the edge of the bed (feet placed on the floor and holding on with the non-affected upper limb), but could not transfer, stand or walk independently. He required the help of at least one person for these tasks. His Functional Ambulation Category (FAC, 0-5, 0 = not able to walk at all, 5 = independent including stair climbing) score was 1, his Rivermead Mobility Index was 3. The lower limb muscle strength was 22, assessed with the help of the Motricity Index (MI, 0-100). His upper limb was plegic, and he was partially dependent in the basic activities of living with a Barthel Index (0-100) of 25.

His comprehensive rehabilitation programme, which had started two weeks earlier, included regular physio- and occupational therapy. Tasks repetitively practiced were the transfer into the wheelchair, sitting balance on the bench, standing with physical help or in a standing frame, and making the first steps alongside the bench with the help of two therapists.

To start the locomotor therapy, the team then decided to additionally use the G-EO-Systems every workday for a total of 25 sessions à 25 - 30 min. During the first five sessions, the harness-secured patient practiced simulated floor walking at a velocity of 0,25 m/sec, the step length (cadence) was 37,5 cm (40 steps/minute). The relative body weight support was 30%. One therapist helped with donning and doffing, which each took three to five minutes, and with manually assisting the knee extension of the paretic side throughout the session. Including breaks due to exhaustion (the maximum heart rate not to exceed was set at 120 beats/min), the net training time was approximately 15 min totalling 600 steps per session in the first week. During the next three weeks, the BWS was gradually reduced to 10% BWS, and the speed increased to 0,33 m/sec during the floor walk condition, manual assistance of knee extension was no longer required. Furthermore, the subject also practiced stair climbing up for a net of five to eight minutes during each session. The cadence started at 35 steps/min, the step rise (step run) was 18 (30) cm. Over the three weeks the cadence was gradually increased to a value of 48 steps/min, the other parameters remained unchanged. Initially the training of stair climbing had to be stopped every two to five minutes as the heart rate exceeded the level of 120 beats/min, in the last week he sustained eight minutes stair climbing training without a break. Physical assistance with knee extension of the paretic side was required throughout the training of stair climbing.

Subjectively, he rated the locomotor training positive but demanding, particularly during the simulated stair climbing. He noticed a constant improvement in his mobility and corporal fitness, a view shared by his therapists.

At the end of the five week period he was able to transfer, and to stand up independently. While standing he had to hold on with the non-paretic hand, and, with the help of a quadricane, he could walk a distance of 20 m without physical support, corresponding to a FAC value of 4. For climbing one flight he needed the physical assistance of one therapist. The lower limb muscle strength was 59 (initial value 22), assessed again with the help of the Motricity Index (0-100), his Rivermead Mobility Index (0-15) increased to 7 (initial value 3) and the Barthel Index (0-100) up to 65 (initial value 25). Table 3 shows the clinical parameters of the present clinical case report.

**Table 3:** Clinical Data of a single case patient before and after the therapy on the G-EO- Systems.

Age		72	
			
Sex		male	
			
Diagnosis		supratentorial, ischemic stroke	
			
Hemiparesis		left	
			
			
	initial value		final value
			
FAC	1		4
			
RMI	3		7
			
MI	22		59
			
BI	25		65

The assessed parameters at the beginning and at the end of the treatment period were the Functional Ambulation Category (FAC, 0-5), the Rivermead Mobility Index (RMI, 0-15), the Motricity Index (MI, 0-100) and the Barthel Index (BI, 0-100). Note an improvement of all the clinical parameters after five weeks of treatment on the gait robot.

## Discussion

The article presents a newly developed gait robot to intensify the gait rehabilitation including stair climbing of stroke patients. The dynamic EMG of selected lower limb muscles of six ambulatory hemiparetic subjects confirmed rather comparable muscle activation patterns during the real and simulated walking on the floor, and a more timely correct pattern of the shank muscles during the simulated stair climbing on the machine as compared to the real walking condition.

The G-EO-Systems followed the intention of the HapticWalker, but specifications included smaller dimensions and an energy supply of 230 V to ensure a maximum clinical applicability. The device met the specifications, however at the price of a reduced maximum acceleration of 1 g during push-off and a reduced maximum speed of 2,3 km/h when compared to the HapticWalker. Those limitations were tolerated, as the primary purpose of the G-EO-Systems was the treatment of gait-impaired stroke patients.

Compared to the technical features of the Lokomat, the currently most applied gait machine, force feedback and a virtual reality application have not yet been realized. Further, an exoskeleton-based system supports the knee during the stance phase, whereas an end-effector based machine may require manual assistance or a functional electrical stimulation of the quadriceps, depending on the grade of paresis, to stabilize the knee during the stance phase. This may be of particular importance in severely affected spinal cord injury patients.

Hidler et al. [24] had studied the activation pattern of healthy subjects on the treadmill and on the Lokomat. The authors observed significant differences in the spatial and temporal muscle activation patterns across various portions of the gait cycle between treadmill and robot-assisted walking, a finding partly confirmed for the LOPES [27], another exoskeleton-based system. Activity in the quardiceps and hamstrings was significantly higher during the swing phase of Lokomat walking than treadmill walking, while activity in the ankle flexor and extensor muscles was reduced throughout most of the gait cycle in the Lokomat.

The present study assessed the dynamic EMG of hemiparetic subjects which renders any comparison with those of healthy subjects difficult given the fact the muscle activation pattern of hemiparetic subjects can vary considerably [28]. The authors therefore concentrated on the intra-individual comparison across real and simulated walking conditions, a statistical comparison was only meaningful in the case of a uniform change induced by the machine.

Unanimously, the onset (duration) of the activation of the quadriceps muscles was delayed (prolonged) throughout the whole stance phase on the G-EO-Systems during the floor walking condition. A less hard impact during the initial contact requiring less stabilization force during the initial contact, and the lack of a metatarsal joint impeding the tibia advancement requiring a prolonged activation may have been the principal cause. For the shank muscles, the activation patterns were rather comparable during both floor walking conditions, the individual patterns varied considerably in line with the types described by Knuttson and Richards [29], either an extremely low muscle activity, or in the sense of a pathological coactivation during part of the gait cycle, thus disrupting the normal sequential shift of activity in antagonistic muscles. During the real stair climbing, the individually disturbed activation pattern of the shank muscles remained, whereas it became more physiological during the simulated stair climbing on the machine in three patients. The coactivation was replaced by an alternating and timely correct pattern. A potential explanation is the more physiological movement on the machine, as the programmed trajectories rendered the gait perfectly symmetric thus prolonging the stance phase of the affected leg on the machine as compared to the real conditions. At the same time, the patients were instructed and assisted by the therapist to flex the hip and knee in the pre-swing and to propel the body upward by a strong activation of the shank muscles. Further, the harness provided safety.

Contrary to the findings with exoskeleton-based systems in healthy subjects [24], the thigh muscles of the hemiparetic subjects did not become active during the swing phase on the G-EO-Systems, confirming the results of the HapticWalker [30]. Of course, any comparison between healthy and hemiparetic subjects is questionable, but one may speculate that the hemiparetic subjects had not to counterbalance any additional inertia or movement restriction imposed by an exoskeleton.

The single case report of a non ambulatory patient does not allow any conclusions on the effectiveness of the gait robot, which was applied additionally to his conventional programme. The patient regarded the training positive, the body weight support (net treatment time) was constantly reduced (extended), and his gait functions improved. Relevant side effects, such as acute arthritis of the lower limb joints or of cardiovascular origin, did not occur.

## Conclusions

The new gait robot enables stroke patients the repetitive practice of not only walking on the floor but also stairs climbing up and down. Major deviations of the lower limb muscle activation patterns of six ambulatory patients did not occur while practising on the machine, rather selected patients exhibited a less coactivated pattern of the shank muscles during the simulated stair climbing up. Any fears of the practice of a less physiological muscle activation pattern on the machine in stroke patients are thus not warranted. The positive single case report does not allow any conclusions on the effectiveness of the device; it will help to design future clinical studies, urgently needed.

## Competing interests

Reha-Technologies GmbH, Bozen, Italy holds the international patent on the device presented. The author SH is a shareholder of the company.

## Authors' contributions

SH conceived the device and drafted the article. AW supported the draft of the article and the analysis of the gathered data. CT worked on the data acquisition, the subsequent signal processing, and helped in the draft of the present manuscript. All authors read and approved the final manuscript.

## Consent

Written informed consent was obtained from the patient for publication of this case report and accompanying images. A copy of the written consent is available for review by the Editor-in-Chief of this journal.
